# Trends in the Antimicrobial Resistance Pattern of Bacterial Gram-Negative Pathogens in Elderly Patients Admitted to the Intensive Care Unit

**DOI:** 10.3390/microorganisms13102330

**Published:** 2025-10-09

**Authors:** Andreea-Loredana Golli, Ovidiu Mircea Zlatian, Simona-Georgiana Popa, Flavia Liliana Turcu, Andrei Theodor Balasoiu

**Affiliations:** 1 Department of Public Health and Management, University of Medicine & Pharmacy Craiova, 200349 Craiova, Romania; andreea.golli@umfcv.ro; 2 Department of Microbiology, University of Medicine and Pharmacy of Craiova, 200349 Craiova, Romania; ovidiu.zlatian@umfcv.ro; 3 Department of Diabetes, Nutrition and Metabolic Diseases, University of Medicine & Pharmacy Craiova, 200349 Craiova, Romania; 4 Department of Nephrol & Dialysis, Carol Davila University of Medicine & Pharmacy Bucharest, 050474 Bucharest, Romania; 5 Department of Ophthalmology, University of Medicine and Pharmacy of Craiova, 200349 Craiova, Romania

**Keywords:** multidrug resistance, Gram-negative pathogens, elderly patients

## Abstract

Antimicrobial resistance (AMR) remains a public health problem in European Union countries, and elderly patients represent a vulnerable category due to aging and its associated risk factors. In this research, we investigated the trend of the antimicrobial resistance pattern of Gram-negative pathogens isolated in samples collected from elderly patients (over 65 years) hospitalized in the intensive care unit (ICU) between 2022 and 2024. A total of 2510 samples, including blood, tracheal aspirate, sputum, urine, pus/wound swabs, exudates, intravascular catheters, cerebrospinal fluid, and sterile fluids, were collected from 1864 elderly patients. Almost two-thirds of clinical specimens were harvested from the respiratory tract. The most frequently reported pathogens from 3094 Gram-negative bacterial isolates were *Klebsiella* spp., *Acinetobacter* spp., and *Escherichia coli*. During the studied period, almost 40% of all the *Klebsiella* spp. strains were multidrug-resistant (MDR)/extensively drug-resistant (XDR), with a significant increase in the resistance to cephalosporins (*p* ≤ 0.05), fluoroquinolones (*p* ≤ 0.05), and carbapenems (imipenem—(*p* ≤ 0.05), ertapenem—*p* < 0.001). The proportion of carbapenem-non susceptible *Klebsiella* spp. rose from 24.41% in 2023 to 32.48% in 2024, *p* = 0.01. Two-thirds of *Acinetobacter* spp. isolates were MDR/XDR, and over 80% were carbapenem-non-susceptible in 2023–2024. The results draw attention to the need to quickly adopt measures to reduce the prevalence, limit the transmission of MDR/XDR pathogens, and improve therapeutic protocols in this age category.

## 1. Introduction

Antimicrobial resistance (AMR) remains a significant concern within the EU/EEA, especially because of the rising rates of carbapenem resistance observed in *Klebsiella pneumoniae* and *Acinetobacter* species between 2020 and 2021, as well as the consistently high levels of antimicrobial resistance reported for *Acinetobacter* spp.) [1]. The majority of isolates resistant to carbapenems were identified among ICU (intensive care unit) patients. In July 2022, the European Commission and the Member States identified AMR as one of the top three priority health threats [2,3]. In early 2024, the World Health Organization (WHO) reported the global emergence of hypervirulent *Klebsiella pneumoniae* (*K. pneumoniae*) ST23 strains harboring carbapenemase genes. The strain was detected in at least one country across all six WHO regions, emphasizing the urgency of robust epidemiological surveillance and genomic monitoring [4].

It is estimated that 39 million deaths will be directly attributed to bacterial AMR between 2025 and 2050 [5].

Antimicrobial resistance (AMR) is a growing concern in elderly patients due to a combination of factors related to aging and healthcare exposure. Several risk factors can contribute to this increased susceptibility. It is estimated that the largest increase in deaths attributable to AMR will be recorded in the case of people over 70 years of age, accounting for 65.9% (61.2–69.8) of all-age deaths attributable to AMR in 2050 [5]. High-burden, drug-resistant pathogens—including carbapenem-resistant Enterobacterales, third-generation cephalosporin-resistant Enterobacterales, and carbapenem-resistant *Acinetobacter baumannii*—are classified among the critical priority group of antibiotic-resistant bacteria, representing the most serious threat to global public health [6].

The excessive or inappropriate use of antibiotics, especially broad-spectrum ones, can drive the development of resistance. Critically ill patients are particularly vulnerable to infections because of several predisposing factors, including immunosuppression, the use of invasive devices (such as central venous catheters, mechanical ventilators, and urinary catheters), extended hospitalizations, and repeated exposure to broad-spectrum antibiotics [7].

The existence of multiple chronic diseases (diabetes, heart failure, and chronic kidney disease) and a compromised immune system make the elderly more susceptible to infections and increase the risk of AMR. Elderly patients with critical conditions admitted to the ICU require invasive medical interventions (urinary catheters, central lines, and mechanical ventilation) and long-term hospitalization, increasing their contact with multidrug-resistant organisms and the risk of contracting hospital-acquired infections. Due to the particularities of this category of patients, these infections have different characteristics regarding clinical manifestations, etiology, treatment outcome, and infection control measures. Conducting studies on antibiotic resistance addressing this vulnerable category allows the identification of possible causes and the establishment of targeted measures. Population aging characterizes all EU countries: on 1 January 2024, older people (aged 65 years and over) had a 21.6% share of the estimated number of 449.3 million people [8]. It is estimated that a quarter of the world’s population will be older than 65 years by 2100 [5], with an expected increase in the global disability-adjusted life years (DALYs) of this age category by 55% between 2004 and 2030 [9].

Romania is one of the EU countries also facing significant demographic challenges due to its rapidly aging population, with 20% of the total population being over 65 years old [8], with a decreasing birth rate and increasing life expectancy, leading to an increasing pressure on the working population and challenges in providing social and medical services for the elderly. This age structure shift contributes to a smaller active population and necessitates policies to manage social security, healthcare, and the labor market effectively to sustain the economy and support the expanding elderly demographic.

Investigating the health condition of the elderly population is essential for understanding the economic objectives within demographic transitions and for developing socioeconomic policies that adequately address the needs of this expanding demographic group [9].

In Romania, infections caused by Gram-negative bacteria that are resistant to last-resort antibiotics represent a major public health concern [10].

Therefore, in the current research, we investigated the trend of the antimicrobial resistance pattern of Gram-negative pathogens isolated in samples collected from elderly patients hospitalized in a tertiary teaching hospital in Romania during a 3-year period (2022–2024).

## 2. Materials and Methods

This research is a retrospective analysis of all the data on cultures collected from elderly patients (over 65 years) admitted to ICU of the Emergency Clinical County Hospital of Craiova, Romania, during 2022 and 2024. This tertiary teaching hospital provides specialized healthcare for complex medical cases from Dolj County and the South-West Region of Romania that cannot be solved at the level of lower-ranking hospitals, especially in the case of emergencies and patients in critical condition. The period under consideration took into account the fact that, since 2022, hospital admissions were based on the necessity of intensive care, following the removal of COVID-19 pandemic measures that had restricted hospitalizations and surgical interventions for patients with chronic conditions.

Data were collected from the clinical pathology databases of the hospital, including samples collected from patients with suggestive signs of infection. Samples collected as part of the hospital’s existing screening protocol were not included. The specimens from elderly patients have been processed in the hospital’s Laboratory of Microbiology.

The analysis included all positive bacterial cultures from elderly patients admitted to the ICU in the studied period, except bacterial duplicates, defined as the same pathogen with the same resistance profile isolated from the same site of infection and the same patient, and samples collected less than 30 days apart, during which the same pathogen was isolated. The samples collected on the same day, from the same patient and from the same site, which highlighted different resistance profiles were also eliminated.

Bacterial isolates from blood, urine, sputum/tracheal aspirate (respiratory secretion), pus/wound swabs, exudates, intravascular catheters, cerebrospinal fluid, and sterile fluids were collected. Blood samples were collected in specialized bottles provided with the automated system Bact/Alert 70^®^ 3D, harvesting a set of two culture bottles for each patient, including one bottle for aerobic bacteria and one for anaerobic bacteria.

The percentage of multidrug-resistant (MDR), extensively drug-resistant (XDR), and pan-drug-resistant (PDR) pathogens were analyzed. An isolated microorganism was defined as multidrug-resistant if the acquired non-susceptibility was demonstrated in at least one agent in three or more antimicrobial categories [11]. For the analysis of multidrug resistance, non-susceptibility to at least three distinct classes of antibiotics—aminoglycosides, cephalosporins, carbapenems, tetracyclines, or fluoroquinolones—was considered [11]. XDR was defined as non-susceptibility to at least one agent in all but two or fewer antimicrobial categories, meaning that bacterial isolates remained susceptible to only one or two classes [11], and PDR was defined as non-susceptibility to all agents in all antimicrobial categories tested in the hospital [12].

The Vitek 2 Compact system (Biomerieux, Durham, NC, USA) was used to identify the Gram-negative isolates (GN cards) and their antibiotic resistance profiles [13]. The antimicrobial susceptibility test was carried out according to Clinical Laboratory Standard Institute (CLSI) guidelines [14]. For all antibiotic classes except polymyxines we used the following Vitek2 cards: AST-N233 and AST-XN05 (for extended antibiogram) for antibiotic susceptibility testing by MIC. The resistance mechanisms as a production of ESBL or carbapenemase were reported by the Vitek system (software version 9.02.4.531). The colistin resistance was tested using the broth microdilution method using the ComASP Colistin Test Panel (Liofilchem, Roseto degli Abruzzi, Italy).

Data from the hospital information system, including patients’ age, sex, hospital department, sample type, site of infection, and antimicrobial resistance pattern of the Gram-negative isolates, were analyzed using Microsoft Excel (2024). The distribution of the Gram-negative microorganisms was analyzed and expressed as percentages. The resistance rates were expressed as the percentage of resistant Gram-negative isolates among all tested Gram-negative isolates. In order to evaluate the trend of antibiotic resistance over the study period, the comparison between different years of bacterial detection and drug resistance rates of pathogens was made, using the chi-square test for independence, or Fisher’s exact test for small groups. Epi Info software, version 7.2.4.0, was used for all statistical analyses. A two-sided *p*-value ≤ 0.05 was considered to be statistically significant.

## 3. Results

### 3.1. Distribution of the Main Isolates

This research included 2510 samples collected between 2022 and 2024 from 1864 elderly patients, aged 65 years or above, admitted to the ICU. More than half of the patients were females (1046—56.12%). The number of patients admitted in 2024 was almost equal to the number of those hospitalized in previous two years (Table 1).

The clinical specimens included blood, tracheal aspirate, sputum, urine, pus/wound swabs, exudates, intravascular catheters, cerebrospinal fluid, and sterile fluids. Among them, 3094 Gram-negative bacterial pathogens have been identified, excluding cases where there was more than one isolate of the same pathogen from the same patient.

During the study period, almost two-thirds of the samples were collected from the respiratory tract—tracheal aspirate and sputum (1564—62.31%), followed by those from blood (363—14.46%), urine (297—11.83%), and pus/wound swabs (190—7.57%). In 2023, the number was nearly twice as many as in the previous year, the increasing trend maintained itself in the following year.

**Figure 1:**
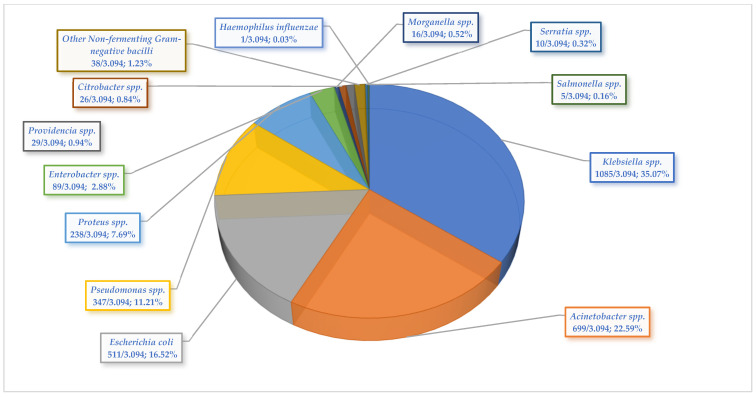
Distribution of the specimens collected from elderly patients hospitalized in the ICU, from 2022 to 2024.

In 2023, the study showed a significant decline in the proportion of blood cultures (*p* < 0.05) and a significant increase in the proportion of the pus/wound swabs (*p* < 0.001) compared with 2022. The percentage of urine samples significantly decreased in 2024 compared with the previous year (Figure 1). The rates of blood and respiratory tract cultures showed an increasing trend in 2024, but it was without statistical significance (Figure 1).

**Figure 2:**
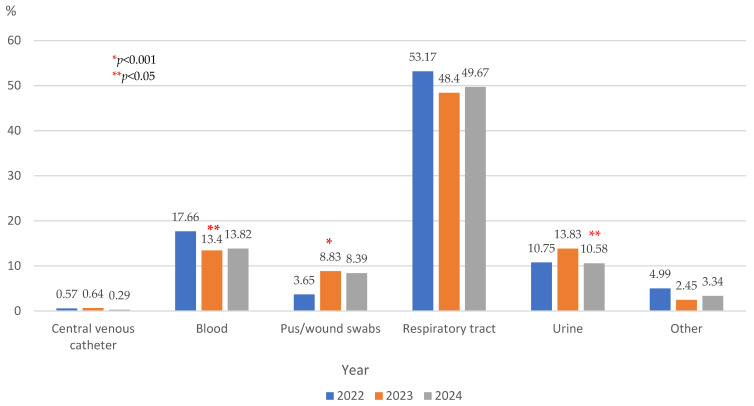
Distribution of Gram-negative pathogens isolated from all specimen types collected from elderly patients hospitalized in the ICU, from 2022 to 2024.

One third of the Gram-negative strains isolated during the entire period analyzed were *Klebsiella* spp. (1085/3094; 35.07%), followed by *Acinetobacter* spp. (699/3.094; 22.59%), and *Escherichia coli* (511/3.094; 16.52%), together accounting for almost 75% of the total Gram-negative bacterial species detected between 2022 and 2024 (Figure 2).

The evolutionary trend of the prevalence of Gram-negative pathogens identified during the study period was analyzed. In the case of *Klebsiella* spp. strains, there was an upward evolution of the prevalence, but the increase was not statistically significant. The positive rates of *Acinetobacter* spp. significantly decreased in 2023 (228/1099; 20.75% compared to the previous year (167/616; 27.11%, *p* = 0.003), the following year being marked by insignificant growth (304/1379; 22.05%). A significant decrease in infection with *Pseudomonas* spp. was found in 2024 (133/1379; 9.64%), compared to the value registered in 2023 (138/1099; 12.56%) (*p* = 0.02). In the case of the positive rates of the other identified pathogens, the variations recorded were not significant (Table S1).

**Figure 3:**
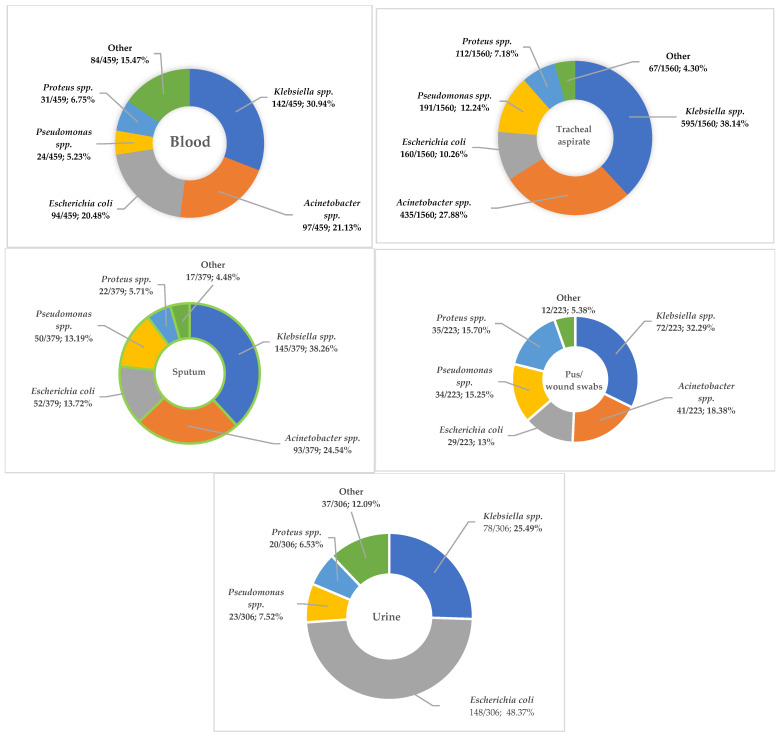
The percentage of the main bacterial species isolated from different sample types (blood, tracheal aspirate, sputum, pus/wound swabs, and urine) of patients over 65 years, from 2022 to 2024.

Relative to the specimen type, *Klebsiella* spp. represented almost 40% (740/1939) of the strains isolated in samples collected from the respiratory tract, also showing the highest prevalence in blood samples (142/459; 30.94%) and in pus/wound swabs (72/223; 32.29%) (Figure 3). *Escherichia coli* was the Gram-negative pathogen isolated with the highest frequency in urine samples (148/306; 48.37%).

### 3.2. Antimicrobial Resistance in Main Bacterial Gram-Negative Species

#### 3.2.1. Characteristics of the Main Gram-Negative Pathogens 

##### *Klebsiella* spp.

*Klebsiella* spp. was the most frequently isolated pathogen in the samples collected from patients over 65 years of age admitted to the ICU, accounting for 35% of the total detected pathogens. During the period analyzed there was a constant, but not significant, increase in the prevalence of this pathogen. Almost 70% of the strains (740/1085) were identified in samples from the respiratory tract and around 13% (142/1085) were from blood.

**Figure 4:**
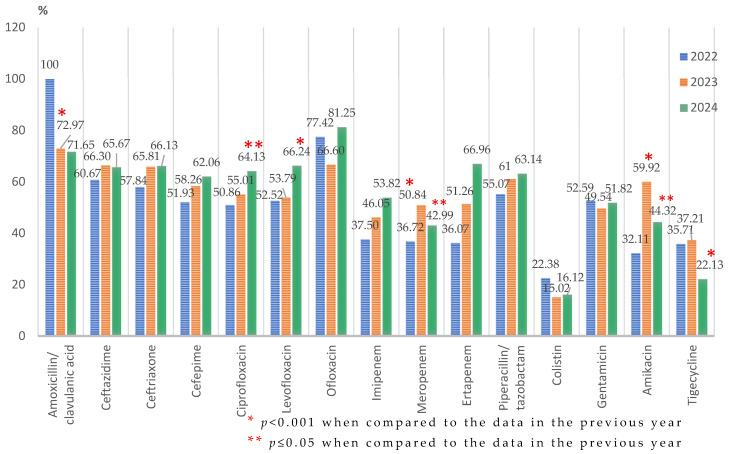
Antimicrobial resistance profile of *Klebsiella* spp. isolated from elderly patients admitted into the ICU, from 2022 to 2024.

The analysis of the percentages of the *Klebsiella* spp.-resistant strains identified throughout the entire study period showed a high resistance to cephalosporins, with 65% of the strains isolated in our study being resistant to second-generation and third-generation cephalosporins and almost 60% to fourth-generation cephalosporins. For the entire studied period, the drug resistance of *Klebsiella* spp. against ofloxacin was the highest (159/209; 76.07%), exceeding 80% (91/112) in 2024. Around 60% of the strains were found to be resistant to fluoroquinolones, 75% to amoxicillin/clavulanic acid, and almost half to carbapenems. In 2023, the research highlighted a significant decrease in the drug resistance rates of the *Klebsiella* spp. against amoxicillin/clavulanic acid (270/370; 72.97%) compared to the previous year, in which all the tested strains were resistant (110/110; 100%), (*p* < 0.001). In the same period, it registered a significant increase in the resistance rate against amikacin (58.92% (66/112) compared to 32.11% (35/109), *p* < 0.001) and meropenem (50.84% (182/358) compared to 36.72% (65/177), *p* = 0.002. However, in 2024, the rate of resistance was significantly reduced in both meropenem (184/428; 42.99%, *p* = 0.03) and amikacin (172/388; 44.32%, *p* = 0.008), but the values were higher than those recorded at the beginning of the interval. In the same year, the resistance to tigecycline (81/366; 22.13%) was significantly lower (*p* < 0.001) than the previous year (48/129; 37.21%), while the resistance has increased significantly for ciprofloxacin from 55.01% (192/349) to 64.13% (320/499), (*p* ≤ 0.05) and for levofloxacin from 53.79% (156/290) to 66.24% (310/468), (*p* < 0.001). (Figure 4).

From 2022 to 2024, a significant increase in the resistance rate of the *Klebsiella* spp. strains to ceftriaxone, cefepime, ciprofloxacin, imipenem, levofloxacin, amikacin (*p* ≤ 0.05), and ertapenem (*p* < 0.001) was observed. A significant decreasing trend during the study period was found only against amoxicillin/clavulanic acid and tigecycline (*p* < 0.001).

##### *Acinetobacter* spp.

*Acinetobacter* spp. ranked second in frequency, representing around 20% of all the Gram-negative strains (699/3094). The year 2023 was characterized by a significant decrease in the percentage of isolated *Acinetobacter* spp. strains [20.75% (228/1099), versus 27.11% (167/616), *p* = 0.003], followed by an upward trend in 2024 (304/1379; 22.05%). During the three-year period, the *Acinetobacter* spp. strains showed a very high level of resistance for the third- and fourth-generation cephalosporins (90–95%), fluoroquinolones (93%), carbapenems (92%), aminoglycosides (81–88%), and piperacillin–tazobactam (92%) (Table S2). Almost 84% of the tested strains were susceptible to colistin.

**Figure 5:**
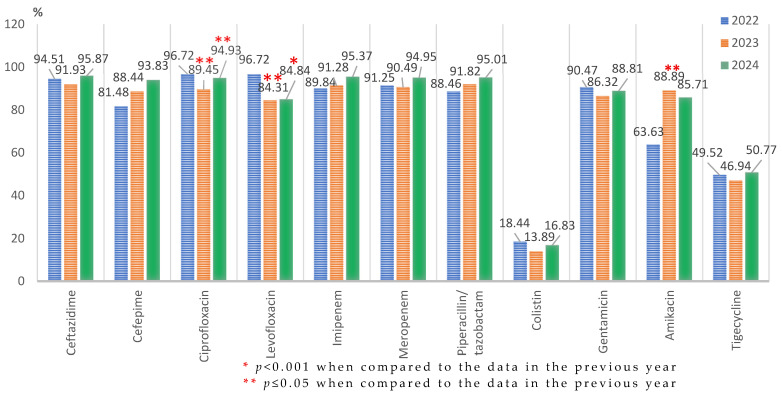
Antimicrobial resistance profile of *Acinetobacter* spp. isolated from elderly patients admitted into the ICU, from 2022 to 2024.

The resistance rate to ciprofloxacin showed a significant decrease in 2023 (195/218; 89.45%) compared to 2022 (118/122; 96.72%, *p* ≤ 0.05), but the trend was significantly reversed in the following year (281/296; 94.93%, *p* ≤ 0.05). The percentages of cephalosporin-resistant and carbapenem-resistant strains remained high during the study period, with no significant changes from one year to another. The resistance to amikacin significantly increased in 2023 (160/180; 88.89%, *p* ≤ 0.05) and was maintained at a high level in the following year (almost 86%—144/168) (Figure 5).

For the entire study period, statistically significant increases were only recorded in the case of cefepime (from 81.48% to 93.83%, *p* < 0.001), amikacin (from 63.63% to 85.71%, *p* < 0.001), and piperacillin–tazobactam (from 88.46% to 95.01%, *p* ≤ 0.05). The variations in 2024 compared to 2022 for the other tested antibiotics were not statistically significant.

##### *Escherichia coli* 

**Figure 6:**
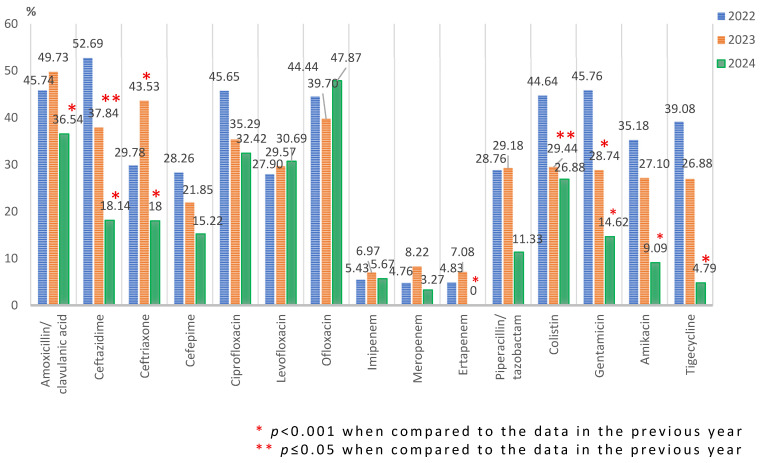
Antimicrobial resistance profile of *Escherichia coli* isolated from elderly patients admitted into the ICU, from 2022 to 2024.

*Escherichia coli* accounted for about 17% (511/3094) of the total identified pathogens, with no significant variations during the study period. Between 30% and 45% of all strains were resistant to fluoroquinolones. Only in the case of ciprofloxacin was there a constant decline in resistance between 2022 (42/92; 45.65%) and 2024 (71/219; 32.42%). For most of the tested antibiotics (amoxicillin/clavulanic acid, ceftazidime, ceftriaxone, aminoglycosides, and tigecycline), there was a sharp decrease (*p* < 0.001) in resistance in 2024, with no resistant strains against ertapenem. A decreasing trend was observed in the case of colistin, which was statistically significant (*p* ≤ 0.05) in 2023 (53/180; 29.44%) compared to 2022 (25/56; 44.64%) (Figure 6).

By analyzing the trend of the resistance rate of the *Escherichia coli* strains during the studied period, we found a significant decrease in 2024 compared to 2022 in the case of most tested antibiotics: cephalosporins (ceftazidime (*p* < 0.001), ceftriaxone (*p* ≤ 0.05), and cefepime (*p* ≤ 0.05)), fluoroquinolones (ciprofloxacin (*p* ≤ 0.05)), piperacillin–tazobactam (*p* < 0.001), colistin (*p* ≤ 0.05), aminoglycosides (gentamicin, amikacin (*p* < 0.001)), and tigecycline (*p* = 0.001) (Figure 6).

##### *Pseudomonas* spp.

**Figure 7:**
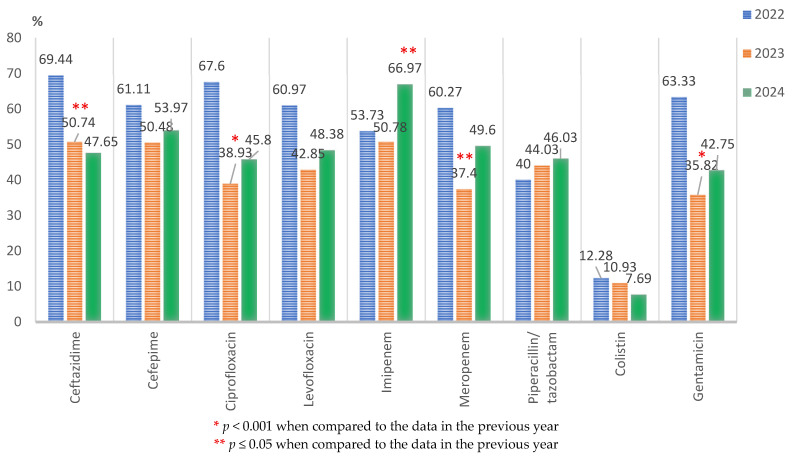
Antimicrobial resistance profile of *Pseudomonas* spp. isolated from elderly patients admitted into the ICU, from 2022 to 2024.

From all the Gram-negative isolated pathogens, *Pseudomonas* spp. strains represented 11.21% (347/3904). During 2022–2024, the highest resistance was identified against tigecycline (112/132; 84.85%), with the case of the other tested antibiotics being around 50%. The resistance rate significantly decreased in 2023 compared to 2022 for ceftazidime (68/134; 50.74%, versus 50/72; 69.44%, *p* < 0.05), ciprofloxacin (51/131; 38.93%, versus 48/71; 67.60%, *p* < 0.001), meropenem (49/131; 37.40%, versus 44/73; 60.27%, *p* < 0.05), and gentamicin (48/134; 35.82%, versus 38/60; 63.33%, *p* < 0.001). Imipenem was the only tested antibiotic for which a significant increase (*p* < 0.05) in resistance was found in 2024 (73/109; 66.97%) compared to 2023 (65/138; 50.78%) (Figure 7).

The resistance rate of *Pseudomonas* spp. strains decreased significantly in 2024 compared to 2022 in the case of ceftazidime, ciprofloxacin, and gentamicin (*p* ≤ 0.05). The changes recorded in the case of the other tested antibiotics had no statistical significance.

##### *Proteus* spp.

**Figure 8:**
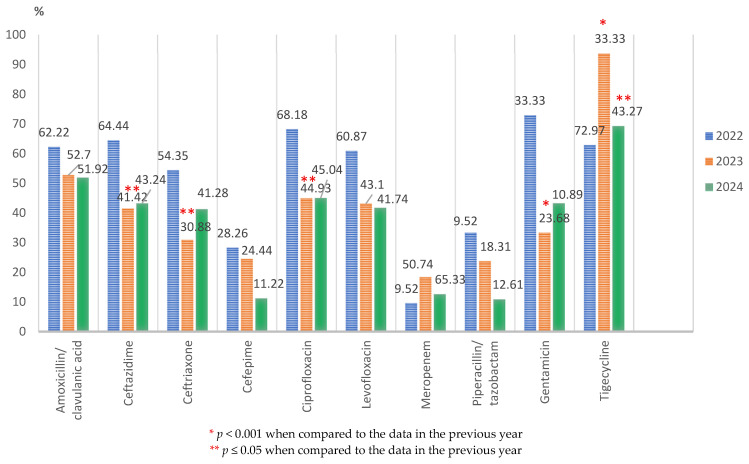
Antimicrobial resistance profile of *Proteus* spp. isolated from elderly patients admitted into the ICU, from 2022 to 2024.

*Proteus* spp. isolates represented 7.69% (238/3094) of all the pathogens. Around half of the tested strains were resistant to imipenem, third-generation cephalosporins, and fluoroquinolones. The resistance rate to tigecycline was the highest for the 3-year period. The drug resistance rates of *Proteus* spp. significantly decreased in 2023 for third-generation cephalosporins (*p* ≤ 0.05), ciprofloxacin (*p* ≤ 0.05), and gentamicin (*p* < 0.001), with no significant changes in the following year, except for piperacillin/tazobactam (*p* ≤ 0.05). (Figure 8). The annual resistance rate significantly increased in 2023 compared to the previous year for imipenem (34/67; 50.74%, versus 11/44; 25%, *p* < 0.05) and tigecycline (45/48; 93.75%, versus 22/35; 62.87%, *p* < 0.001). In 2024, there was a significant decline in the pathogen’s resistance to piperacillin/tazobactam (11/101; 10.89%, versus 18/76; 23.68%, *p* ≤ 0.05) and tigecycline (61/88; 69.32%, *p* ≤ 0.05).

The resistance trend of *Proteus* spp. strains in 2024 compared to 2022 shows a statistically significant decline for ceftazidime (*p* ≤ 0.05), cefepime (*p* ≤ 0.05), ciprofloxacin (*p* ≤ 0.05), piperacillin–tazobactam (*p* ≤ 0.05), and gentamicin (*p* = 0.001) (Figure 8).

Among the Gram-negative isolates tested, colistin MIC values ranged from 0.5 to 16 mg/L, with a mean of 1.74 ± 3.62 mg/L and a median of 0.5 mg/L. *Escherichia coli* strains showed the lowest MICs (median = 0.5 mg/L, mean = 0.52 ± 0.09), while *Pseudomonas* spp. displayed the highest values (median = 2 mg/L, mean = 3.6 ± 5.12), followed by *Klebsiella* spp. and *Acinetobacter* sp., indicating variable susceptibility across species. *Proteus* spp. is intrinsically resistant to colistin.

#### 3.2.2. Multidrug Resistance of the Main Gram-Negative Pathogens

**Figure 9:**
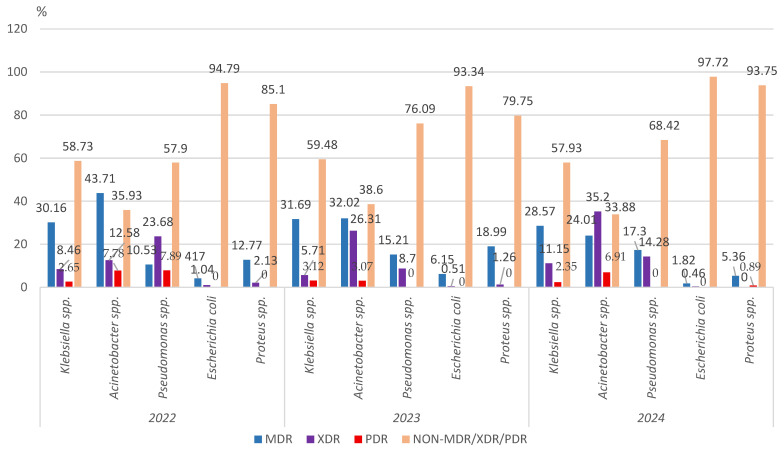
Distribution of the Gram-negative microorganisms isolated from samples from elderly patients hospitalized in the ICU, County Emergency Clinical Hospital Craiova, Romania, 2022–2024; MDR—multidrug-resistant; XDR—extensively drug-resistant; PDR—pan-drug-resistant.

By analyzing the prevalence of multidrug-resistant Gram-negative pathogens identified during the entire study period in the samples collected from elderly patients, we found that almost 40% (420/1085) of all the *Klebsiella* spp. strains were MDR/XDR and almost 3% (29/1085) were PDR (Figure 9).

Approximately 60% of the *Acinetobacter* spp. strains (407/699) were MDR/XDR, registering a constant upward trend during the three years analyzed. The research also highlighted the presence of the 41 PDR pathogens (5.87%), from which 21 were identified in 2024.

Less than 5% (23/511) of the *Escherichia coli* strains were MDR/XDR, and no PDR strain was found.

The prevalence of all the MDR and XDR *Pseudomonas* spp. strains was around 30%, the lowest prevalence being recorded in 2023 (23.91%). In 2022 there were six PDR strains.

Around 12% of the *Proteus* spp. strains were MDR/XDR (29/238), the percentage halved in 2024 compared to the beginning of the study period. One PDR strain was identified in 2024 (Figure 9).

The comparative analysis showed no statistically significant difference in the percentage of MDR/XDR Gram-negative strains between 2022 and 2024.

#### 3.2.3. Carbapenem Non-Susceptibility

**Figure 10:**
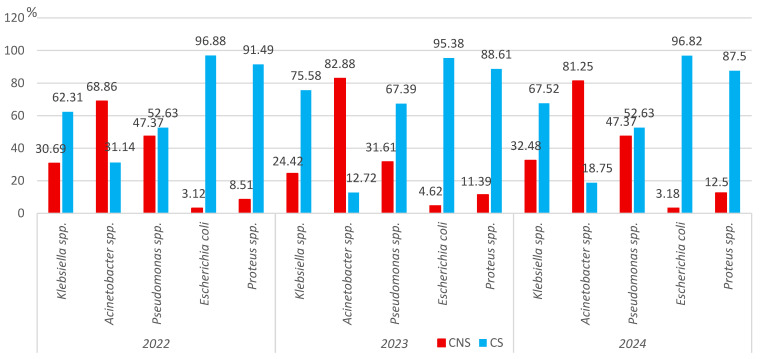
Distribution of the main carbapenem-non-susceptible microorganisms isolated from samples from elderly patients hospitalized in the ICU, County Emergency Clinical Hospital Craiova, Romania, 2022–2024. CNS—carbapenem-non-susceptible; CS-carbapenem-susceptible.

We analyzed the prevalence of carbapenem-non-susceptible (CNS) Gram-negative pathogens isolated in positive cultures of hospitalized elderly patients. The proportion of CNS *Klebsiella* spp. increased significantly in 2024 (166/511; 32.48%) compared to the previous year (94/385; 24.41%, *p* = 0.01), being slightly higher than at the beginning of the study period (58/189; 30.69%). The proportion of CNS *Acinetobacter* spp. strains significantly increased in 2023 (189/228, 82.88%) compared to 2022 (115/167; 68.86%, *p* < 0.001), decreasing slightly in the following year (247/304; 81.25%) (Figure 10). In the case of CNS *Pseudomonas* spp. strains, the proportion significantly declined in 2023 (45/138; 31.61%, *p* < 0.05), reaching in the following year the same value as in 2022 (36/76; 47.37%, *p* < 0.05). About 3% of *Escherichia coli* strains were CNS in 2022 (3/96) and 2024 (7/220), with a slightly higher value (9/195; 4.32%) being recorded in 2023. The proportion of CNS *Proteus* spp. strains ranged from 8.51% (4/47) to 12.5% (14/112), but the difference was not significant (Figure 10).

## 4. Discussion

The proportion of elderly individuals is steadily increasing in developed countries, with older adults accounting for approximately 60% of all ICU stays [15]. Geriatric patients are more susceptible to being infected by multidrug-resistant Gram-negative organisms, including *A. baumannii*, ESBL-producing Enterobacteriaceae, and carbapenem-resistant Enterobacteriaceae [16], having a 1.31-fold increased likelihood of developing sepsis due to Gram-negative bacteria [15]. Due to their treatment complexity, these infections are associated with elevated mortality rates, increased length of hospital stay, and greater healthcare expenditures [17].

Addressing this issue requires a multifaceted approach, including optimizing antibiotic use, developing new therapies, and improving infection prevention and control measures.

Considering the fact that critically ill elderly patients represent a population at risk for infection by MDROs, carrying out research to identify the highly prevalent MDR Gram-negative pathogens is of great importance for the development of new strategies to prevent the acquisition of these strains among the elderly.

However, currently, there is a lack of epidemiological data on the prevalence of MDR infections in the elderly population, supporting the development of preventive strategies against multidrug-resistant bacterial infections in elderly patients.

In the present retrospective observational study, we aimed at detecting the main Gram-negative pathogens involved in infections diagnosed in elderly patients hospitalized in the ICU in a tertiary teaching hospital, over a 3-year period. Almost 80% of the positive samples were identified in 2023–2024, correlating with the end of the epidemiological alert period.

Gram-negative infections were predominantly located in the respiratory tract, the most common isolated strains being *Klebsiella* spp. (35.07%), followed by *Acinetobacter* spp. (22.59%), and *Escherichia coli* (16.52%). A similar percentage of *Acinetobacter* spp. strains has been identified in other investigations, but this pathogen has been implicated in the etiology of most infections [18,19,20]. These studies have highlighted a high percentage held by *Klebsiella* spp. and *Pseudomonas aeruginosa* in the etiology of infections detected in patients hospitalized in the ICU [18,19] while *Pseudomonas aeruginosa* (23.5%) was the most common organism in ICU isolates in research conducted in 2018–2020 in United States hospitals [21].

In a study conducted in long-term care facilities in Italy, urinary tract and lower respiratory tract infections were the most common, mostly being determined by Gram-negative pathogens, highlighting their growing significance among the elderly [22]. A two-center retrospective cohort study of adult hospitalized patients conducted by Narayanan et al. found that among older adult hospitalized patients, obesity was independently associated with the presence of a Gram-negative MDR bacteria (presumptive ESBL or CRE) in a culture [23]. This finding draws attention to the fact that obesity is an additional risk factor that must be taken into account in the development of strategies to combat antibiotic resistance in elderly patients, in view of the fact that obesity continues to be a major health problem with epidemic proportions anywhere in the world, with between 10% and 30% of European Union adults being obese [24].

According to the data published by the European Centre for Disease Prevention and Control, in 2023, Romania was one of the European Union countries who reported the highest resistance of invasive strains of *Klebsiella pneumoniae* to third-generation cephalosporins (more than 50%) [10]. Also, Romania was 1 of 15 countries that reported AMR percentages equal to or above 50% [1] and one of the South-Eastern European countries with a much higher risk of selecting MDR/XDR bacteria (55.1%) than the European average (38.6%) [25].

The results obtained from the research we have carried out revealed that, between 2022 and 2024, around 60% of the *Klebsiella* spp. strains were resistant to cephalosporins and fluoroquinolones and almost half to carbapenems, with a significant increase in antimicrobial resistance among these classes of antibiotics. A significant resistance to cephalosporins, in the case of the *Klebsiella* spp. strains, was found also in other studies conducted in Romania [26,27,28] and in other countries [29,30,31].

These data are consistent with national surveillance showing high carbapenem resistance, more than half of the invasive strains of *Acinetobacter* spp. and *Klebsiella pneumoniae* were found to be resistant to carbapenems (imipenem/meropenem) [10]. These findings show that Romania must make additional efforts to be able to reach the targets recommended by the Council of the EU regarding the reduction in carbapenem-resistant *K. pneumoniae* by 5% by 2030 against the baseline year 2019. These targets aim, in the case of Romania, to reduce third-generation cephalosporin-resistant *E. coli* by 5% against the baseline year 2019 [32].

The proportion of *Klebsiella* spp. MDR/XDR strains was almost 40%, consistent with the reported one from a study regarding Gram-negative bacteria isolated in surgical ICUs in the largest medical center in Croatia [33].

Our study revealed a high percentage of the MDR/XDR *Acinetobacter* spp. strains and a significant increase in the antibiotic resistance rate against cefepime, amikacin, and piperacillin–tazobactam. Over 80% of the strains were carbapenem-non-susceptible in 2023–2024, significantly higher compared with 2022. These values are consistent with those reported at the national level. According to the ECDC Annual Epidemiological Report, in 2023, Romania was one of the five EU countries with the highest percentage of invasive carbapenem-resistant strains of *Acinetobater* spp., with over 50% of the isolated strains being resistant to imipenem/meropenem [10]. The results are also correlated with previous reports [27,28].

This raises serious concern because carbapenem-resistant bacteria are considered an urgent public health threat [34] due to their ability to cause severe infections and their potential to spread rapidly in healthcare settings and due to the fact that they are associated with difficulties in the treatment of these infections and increased mortality [35].

According to the WHO, carbapenems are classified as critically important antimicrobials for human medicine [36], being the third most commonly used class of antibiotics worldwide for the treatment of community-acquired infections in the intensive care unit [37].

Carbapenems are widely recognized as the most effective last-line agents for the treatment of bacterial infections caused by Gram-negative pathogens multi-resistant to antibiotics, especially ESBL-producing Enterobacteriaceae or non-fermentative bacilli: *P. aeruginosa* and *A. baumannii*. [38] *Acinetobacter baumannii* represents one of the most prevalent Gram-negative pathogens linked to hospital-acquired infections worldwide, being responsible for up to 20% of the infections reported in intensive care units [39].

The high percentage of carbapenem-non-susceptibility strains highlighted in our research draws attention to the dramatic limitation of the possibilities of treating the infections produced by these pathogens. This can be due to the fact that carbapenem-non-susceptible Gram-negatives exhibit resistance to all or nearly all β-lactam antibiotics and frequently carry genes encoding for resistance mechanisms against fluoroquinolones and/or aminoglycosides [40]. Our study also showed that about half of the strains of *Acinetobacter* spp. were resistant to tigecycline, which further limits therapeutic options.

*Escherichia coli* was the Gram-negative pathogen most commonly isolated in urine samples in our study, taking into account the fact that UTI is almost always related to an indwelling urinary catheter and *E. coli* is usually the most common infectious agent. The results were consistent with other studies [41,42,43,44].

During the period analyzed, the research revealed a significant decrease in the resistance rate of *E. coli* strains against most of the tested antibiotics, including third-generation cephalosporins. These observations illustrate that Romania reaches the target recommended by the Council of the EU of a 5% reduction in third-generation cephalosporin-resistant *E. coli* by 2030 against the baseline year 2019 [32].

Our data showed a low resistance rate to colistin for the main Gram-negative pathogens involved in the etiology of infections in elderly patients hospitalized in the intensive care unit. These findings may indicate the result of implementing a judicious antibiotic use policy, particularly in vulnerable patients, reserving last-line agents such as colistin exclusively for severe multidrug-resistant Gram-negative infections lacking alternative therapies, depending on the antibiogram.

This policy is part of the intervention to prevent and control the dissemination of MDR-Gram-negative pathogens adopted in our hospital. The implementation of hand hygiene education programs, contact precautions, active screening culture and isolation of the colonized and infected patients, and environmental cleaning, represent infection control measures to reduce transmission of multidrug-resistant Gram-negative bacteria in hospital patients [45].

The specific strategies aimed at reducing AMR in elderly patients should also include an improvement in antibiotic stewardship programs, in order to monitor possible adverse effects and the appropriate prescribing of antibiotics, adjusting doses in relation to comorbidities (malignancies, hematological disorders, chronic illnesses, malnutrition, diabetes, obesity, and immunodepression). The limitation of the use of the medical devices and invasive procedures among elderly patients, and the compliance with the rules of aseptic insertion, cleaning, disinfection, and sterilization can contribute to the reduction in the risk of associated infections and death. Using new diagnostic technologies like multiplex PCR, MALDI-TOF MS, metagenomics, and point-of-care diagnostics, aimed at rapidly detecting pathogens and resistance mechanisms [7], is a very useful tool both for the early initiation of specific antibiotic treatment and for limiting the transmission of infection and the selection of multidrug-resistant pathogens. However, it is difficult to implement such a strategy especially in low- and middle-income countries, due to the high cost associated with the acquisition and maintenance of these advanced diagnostic technologies. Our study has several limitations related to the retrospective nature of the study and to the fact that it includes data from a single tertiary hospital. Other limitations are related to potential selection bias, clustering of isolates per patient, and missing clinical outcome data. However, the article provides valuable information regarding the antimicrobial resistance of Gram-negative pathogens in critically ill elderly patients, considering the fact that the samples were collected from elderly patients hospitalized in an intensive care unit from one of the largest hospitals in Romania. Also, most of the published studies focused specifically on multidrug-resistant bacteria in older hospitalized patients with urinary tract infections. Further multicenter studies could provide more detailed information regarding multidrug-resistant Gram-negative organisms involved in infections in elderly patients, which can be the basis for the development and improvement of prevention and spread control strategies.

## 5. Conclusions

Our findings revealed a very high resistance rate of the *Acinetobacter* spp. strains to the tested representatives from all classes of antibiotics, with a significant increase in the percentage of the carbapenem-non-susceptible isolates. Similarly, *Klebsiella* spp. strains demonstrated a significant increase in resistance to cephalosporins, fluoroquinolones, and carbapenems.

These results highlight the urgent need for the rapid implementation of strategies aimed at reducing the prevalence of multidrug-resistant pathogens in elderly patients, which must address the factors that contribute to the increased risk of AMR in this category of patients.

Such strategies should include high standards of infection prevention and control in hospitals, the improvement of therapeutic protocols addressed to this age category, and raising awareness among prescribers regarding the judicious administration of antibiotics.

## Figures and Tables

**Table 1:** Distribution by gender of the elderly patients admitted to the ICU, County Emergency Clinical Hospital Craiova, Romania, from 2022 to 2024.

*Patients*	*Year*		
	2022	2023	2024
Male	142 (43.56%)	294 (47.57%)	382 (41.52%)
Female	184 (54.44%)	324 (52.43%)	538 (58.48%)
**Total**	326	618	920

## Data Availability

The original contributions presented in this study are included in the article/Supplementary Material. Further inquiries can be directed to the corresponding author.

## Supplementary Materials

The following supporting information can be downloaded at: https://www.mdpi.com/article/10.3390/microorganisms13102330/s1, Table S1: Distribution of Gram-negative pathogens isolated from critically ill patients, from 2022 to 2024; Table S2: Antimicrobial resistance pattern of the main Gram-negative pathogens isolated from critically ill elderly patients, 2022–2024.
