# Supplementary material for: Trends in the Antimicrobial Resistance Pattern of Bacterial Gram-Negative Pathogens in Elderly Patients Admitted to the Intensive Care Unit
Source: Microorganisms. 2025 Oct 9;13(10):2330. doi: 10.3390/microorganisms13102330 (PMC12566077; doi:10.3390/microorganisms13102330)
Supplement: Supplementary file 1 [file microorganisms-13-02330-s001.zip › table S2.pdf]

Table S2. Antimicrobial resistance pattern of the main Gram-negative pathogens isolated from critically ill elderly patients, 2022–2024.

**Antimicrobial Agent**	***Klebsiella* spp. (n=1085)**	***Acinetobacter* spp. (n=699)**	***Escherichia coli* (n=511)**	***Pseudomonas* spp. (n=347)**	***Proteus* spp. (n=238)**
Amoxicillin/ clavulanic acid	728/969 (75.13%)	-	210/485 (43.29%)	-	121/223 (54.26%)
Ceftazidime	683/1050 (65.04%)	639/678 (94.24%)	158/493 (32.05%)	179/334 (53.59%)	106/229 (46.29%)
Ceftriaxone	666/1032 (64.53%)	535/553 (96.74%)	140/615 (22.76%)	-	91/223 (40.81%)
Cefepime	518/879 (58.93%)	559/627 (89.15%)	82/408 (20.09%)	164/301 (54.48%)	35/189 (18.52%)
Imipenem	353/744 (47.44%)	561/605 (92.72%)	22/362 (6.07%)	174/304 (57.23%)	94/186 (50.54%)
Meropenem	531/1033 (51.40%)	628/678 (92.62%)	23/435 (5.28%)	156/331 (47.13%)	31/224 (13.84%)
Ciprofloxacin	601/1023 (58.75%)	594/636 (93.39%)	173/481 (35.96%)	159/333 (47.74%)	111/224 (49.55%)
Levofloxacin	518/857 (60.44%)	421/454 (92.73%)	116/387 (29.97%)	133/277 (48.01%)	82/184 (44.56%)
Ofloxacin	159/209 (76.07)	-	84/189 (44.44)	-	-
Piperacillin/ tazobactam	599/979 (61.18%)	626/677 (92.46%)	98/461 (21.26%)	145/330 (43.94%)	40/210 (19.04%)
Colistin	165/996 (16.56%)	106/654 (16.21%)	135/448 (30.13%)	31/315 (9.84%)	160/160 (100%)
Gentamicin	491/960 (51.14%)	578/654 (88.38%)	106/438 (24.20%)	142/325 (43.69%)	94/212 (44.34%)
Amikacin	273/609 (44.82%)	226/279 (81.00%)	64/337 (18.99%)	82/197 (41.62%)	-
Tigecycline	194/677 (28.65%)	276/561 (49.19%)	67/347 (19.31%)	-	67/93 (72.04%)

Percentage of each column is calculated by dividing the resistance strains to the tested ones; `-` not tested.
