# Supplementary material for: Trends in the Antimicrobial Resistance Pattern of Bacterial Gram-Negative Pathogens in Elderly Patients Admitted to the Intensive Care Unit
Source: Microorganisms. 2025 Oct 9;13(10):2330. doi: 10.3390/microorganisms13102330 (PMC12566077; doi:10.3390/microorganisms13102330)
Supplement: Supplementary file 1 [file microorganisms-13-02330-s001.zip › Table S1.pdf]

Table S1. Distribution of Gram-negative pathogens isolated from critically ill patients, from 2022 to 2024.

Pathogen	Year			*Total*
	2022 (n=616)	2023 (n=1099)	2024 (n=1379)	(n=3094)
Klebsiella spp.	189 (30.68%)	385 (35.03%)	511 (37.06%)	1085 (35.07%)
Acinetobacter spp.	167 (27.11%)	228 (20.75%)**	304 (22.05%)	699 (22.59%)
Escherichia coli	96 (15.58%)	195 (17.74%)	220 (15.96%)	511 (16.52%)
Pseudomonas spp.	76 (12.34%)	138 (12.56%)	133** (9.64%)	347 (11.21%)
Proteus spp.	47 (7.63%)	79 (7.19%)	112 (8.12%)	238 (7.69%)
Enterobacter spp.	10 (1.62%)	34 (3.09%)	45 (3.26%)	89 (2.88%)
Citrobacter spp.	4 (0.65%)	9 (0.82%)	13 (0.94%)	26 (0.84%)
Providencia spp.	10 (1.62%)	7 (0.64%)	12 (0.87%)	29 (0.94%)
Other Non-fermenting Gram-negative bacilli	14 (2.27%)	17 (1.54%)	7 (0.51%)	38 (1.23%)
Other Gram-negative pathogens	3 (0.49%)	7 (0.64%)	22 (1.59%)	32 (1.03%)

**p<0.05.
